# Factors Influencing Family Health History Collection among Young Adults: A Structural Equation Modeling

**DOI:** 10.3390/genes13040612

**Published:** 2022-03-29

**Authors:** Ming Li, Shixi Zhao, Yu-Yu Hsiao, Oi-Man Kwok, Tung-Sung Tseng, Lei-Shih Chen

**Affiliations:** 1 Department of Health Sciences, Towson University, Towson, MD 21252, USA; mli@towson.edu; 2 Department of Health, Exercise & Sports Sciences, University of New Mexico, Albuquerque, NM 87131, USA; shixizhao@unm.edu; 3 Department of Individual, Family, and Community Education, University of New Mexico, Albuquerque, NM 87131, USA; yuyuhsiao@unm.edu; 4 Department of Educational Psychology, Texas A & M University, College Station, TX 77843, USA; omkwok@tamu.edu; 5 Behavioral and Community Health Sciences Program, School of Public Health, Louisiana State University Health Sciences Center, New Orleans, LA 70112, USA; ttseng@lsuhsc.edu; 6 Department of Health and Kinesiology, Texas A & M University, College Station, TX 77843, USA

**Keywords:** family health history, college students, communication, knowledge, behavior

## Abstract

Family health history (FHH) can serve as an entry point for preventive medicine by providing risk estimations for many common health conditions. College is a critical time for young adults to begin to understand the value of FHH collection, and to establish healthy behaviors to prevent FHH-related diseases. This study seeks to develop an integrated theoretical framework to examine FHH collection behavior and associated factors among college students. A sample of 2670 college students with an average age of 21.1 years completed a web-based survey. Less than half (49.8%) reported actively seeking FHH information from their family members. Respondents’ knowledge about FHH were generally low. Structural equation modeling findings suggested an adequate model fit between our survey data and the proposed integrated theoretical framework. Respondents who were members of racial/ethnic minority groups exhibited higher levels of anxiety and intention to obtain FHH information but had lower confidence in their ability to gather FHH information than non-Hispanic White respondents. Therefore, educational programs designed to enhance the level of young adults’ FHH knowledge, efficacy, and behavior in FHH collection, and change subjective norms are critically needed in the future, especially for these who are members of racial/ethnic minority groups.

## 1. Introduction

Family health history (FHH) is a significant risk factor associated with many common and multifactorial health conditions such as cancer, heart disease, and type 2 diabetes because it can capture genetic, behavioral, and environmental factors associated with these diseases that run in one’s family [1,2]. College is a critical time for young adults aged from 18 to 35 years to seek FHH information from family members for a number of reasons. First, the incidence and earlier onset of many of the chronic diseases related to FHH (e.g., obesity, cancer, and type 2 diabetes) are increasing [3,4,5]. Given that FHH can be used to assess disease risks [6], college students who are unaware of their FHH may not recognize potential health threats and be able to take timely preventive actions. Second, the period in which young people pursue post-secondary education is an ideal time to establish future lifestyle-related behaviors [7]. FHH can provide critical information that can help college students establish healthy behaviors early in life that can result in a significant, positive impact on their future health. Third, the research literature has shown that FHH can motivate individuals to adopt healthier behaviors [8,9,10,11,12,13,14]. Thus, FHH information may encourage college students to improve their levels of exercise and healthy eating, maintain an appropriate weight, and reduce alcohol intake. Fourth, young adults in college are at the ideal time in their lives to quickly learn and apply new knowledge [15], and become more comfortable in sharing vital information in FHH communication [16]. Those who collect and share FHH information may influence other family members to also discuss and collect FHH which will result in the creation of a more comprehensive FHH [16,17]. Lastly, with the current increased application of genetic tests in precision medicine for disease prevention, diagnosis, and treatment [18], FHH information can help physicians to determine the needs of genetic tests for college students.

Although it is important for college students to gather their FHH, few studies have assessed this behavior [17,19,20,21]. A survey study at a university setting [17] showed that a majority of the young adult respondents were aware of FHH, but fewer than 40% had collected it. In another survey study, Smith and colleagues [19] reported that female college students were more likely to seek FHH information and share it with family members than were male students, but they also found that both groups perceived barriers to collect FHH. The other two studies investigated FHH information seeking intention among young adults using the Theory of Motivated Information Management (TMIM). In alignment with TMIM principles, both these studies found that FHH related uncertainty discrepancy (the discrepancy between individuals’ actual and desired level of uncertainty) and associated emotional factors (e.g., anxiety and distress) were linked with young adults’ intention to seek FHH information [20,21]. These findings also suggested that FHH collection was a complex behavior that was impacted by several sociodemographic and psychological factors.

Based upon the results of the studies described above, this study aims to develop and examine an integrated theoretical framework that can be used to assess college students’ FHH collection behavior. Given that FHH collection is a complex behavior [20,21], the development of an integrated theoretical framework encompassing multiple levels (i.e., interpersonal and intrapersonal health behaviors and health communication) has the potential to make a significant contribution to improving our understanding of FHH collection behavior [22]. The purposes of this study are threefold. First, we seek to assess young adults’ behavior in FHH collection from family members. Second, we attempt to examine the psychological factors associated with such behavior using an integrated theoretical framework. Third, we will test if sociodemographic characteristics and FHH knowledge are correlated to FHH collection behavior among young adults.

## 2. Materials and Methods

### 2.1. Participants and Procedures

This study was approved by the Texas A&M University Institutional Review Board. Participation criteria included undergraduate or graduate student enrollment at two campuses of a public research-intensive university, and persons aged 18–35 years to meet the definition of young adult [23]. We used Qualtrics (http://www.qualtrics.com (accessed from 15 October 2018 to 20 November 2018)) to collect survey data. Responses were anonymous and participation was voluntary. All participants who completed the survey were given the opportunity to enter a drawing for one of 40 available $50 electronic gift cards as an incentive to participate. The first 100 participants who completed the survey received an additional $5 electronic gift card. To protect participant privacy, participants who wished to be entered in the drawing for electronic gift card incentives were linked to a separate survey to enter their names and emails for incentives that was not in any way associated with the initial survey. We used the university bulk email service to send the initial recruitment email and three reminder emails with the survey link to 55,295 college students. A total of 2809 students filled out the survey, which yielded a response rate of 5.08%.

### 2.2. Survey Development

**Figure 1:**
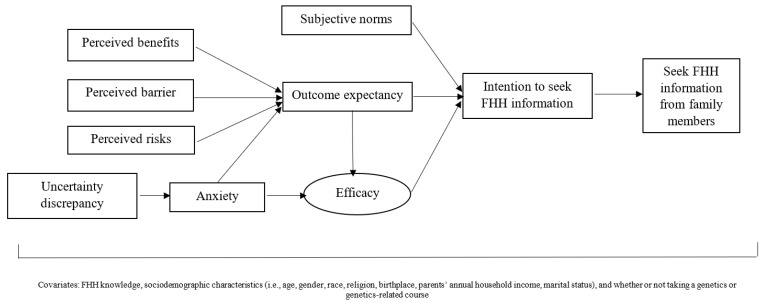
Proposed integrated theoretical model of FHH information seeking behavior among young adults. FHH: family health history.

We developed a 15-min web-based survey based on the integrated theoretical framework and previous literature [19,20,21,24,25,26,27,28,29,30,31]. The constructs in the integrated theoretical framework (Figure 1) were adopted from the key health behavior and communication theories related to FHH collection used in previous studies (i.e., the Health Behavior Model, the Theory of Planned Behavior, and the TMIM) [19,20,21,32]. As presented in Figure 1, subjective norms, outcome expectancy, and efficacy in FHH collection from family members were correlated with young adults’ intention to seek FHH information, which was directly associated to the behavior of FHH collection. Outcome expectancy regarding FHH collection was associated with young adults’ perceptions of the benefits of and barriers to FHH collection, their risk perceptions of developing diseases that run in one’s family, and anxiety resulting from the uncertainties involved in the unknown FHH. Moreover, efficacy was associated with both outcome expectancy and anxiety, which was also linked with uncertainty discrepancy toward FHH information. The definition and detailed measures are described in Table 1. In addition, sociodemographic characteristics (e.g., age, gender, birthplace, race/ethnicity, religion, and marital status), FHH knowledge (Table 2), and whether or not respondents had taken a genetics or genetics-related course as a college or graduate student were measured in the survey and added to the SEM model as moderator variables to determine the effect that those factors had on FHH collection behavior [19,33,34].

### 2.3. Survey Pre-Test

To ensure content validity, experts from multiple related fields including statistics, health education, health communication, college health, and public health genomics reviewed the survey. The survey was then revised based on their feedback. Subsequently, cognitive interviews with a convenience sample of nine college students, and retrospective interviews with additional eight college students were conducted. Minor changes that were made to the survey addressed wording, clarity, and formatting issues. The revised survey was then pilot tested with 63 young adults recruited from two undergraduate classes. The final version of the survey included 16 sections with 95 items. The data collected in the pilot test showed adequate data validity and reliability. We did make minor revisions to the wording of the uncertainty discrepancy items to improve clarity.

### 2.4. Data Analysis Strategies

Survey data cleaning, missingness, descriptive statistics, and psychometric testing (validity using confirmatory factor analysis and reliability using Cronbach’s alpha) were conducted using STATA 15 (Stata, College Station, TX, USA). Missing data analysis was performed to examine any difference between respondents who completed only the demographic information, and those who completed or partially completed the remaining survey [35]. Psychometric testing of each psychological construct showed acceptable data reliability and validity (Figure 1). Bivariate correlations were conducted to examine the relationships between main dependent variables of the psychological constructs (i.e., anxiety, outcome expectancy, efficacy, intention, and behavior in FHH collection) and covariates (i.e., sociodemographic characteristics, FHH knowledge, and whether or not the respondent had taken genetics/genetics-related courses in college). Those covariates with significant bivariate associations with the main psychological constructs were included in the final SEM model. M-plus 8.0 (Muthén & Muthén, Los Angeles, CA, USA) was used to analyze the relationships among the constructs in the proposed theoretical framework [36]. Because chi-square is sensitive to large sample sizes [37], model fit was assessed using three fit indices including the root mean square error of approximation (RMSEA), comparative fit index (CFI), and standardized root mean residual (SRMR). In this study, a RMSEA < 0.08, a CFI > 0.90, and a SRMR < 0.06, were adopted as the cut-off points for an adequate model fit [38].

## 3. Results

### 3.1. Demographic Characteristics

We excluded the responses of 139 participants who completed only the sociodemographic information portion of the survey from the final sample. The final sample consisted of 2670 young adults with the average age of 21.0 years (SD = 3.4, range = 18–35). A majority of respondents were female (66.3%) and born in the United States (78.4%). Approximately half were self-identified as non-Hispanic White (44.9%). Nearly two-thirds of the participants (64.3%) practiced Christian. About one fourth (23.2%) reported no religious affiliation, and the remaining 12.5% practiced other religions, such as Hinduism, Muslim, and Buddhism. About half of participants reported that they had taken a genetics course in college (15.0%) or were currently or previously enrolled in a course containing genetics-related information (32.7%). We used six true/false items to measure FHH knowledge and respondents’ average correct rate was 57.8%. Table 2 presents the percentage of correct answers for each FHH knowledge item. Moreover, the mean score (5.2 ± 1.3) was high for the participants’ perception of importance of FHH collection, which suggested that respondents believed that seeking FHH information from their family members was important. As issue importance is a necessary condition of the TMIM, the high mean score indicates that the condition was met in our sample.

### 3.2. Behavior in FHH Collection with Family Members

Slightly less than half (49.8%) reported actively sought FHH information from their family members. The remaining participants in our sample reported that they had never, rarely, or occasionally sought FHH information from their family members during the past six months.

### 3.3. Psychological Factors Associated with FHH Collection Behavior: SEM Findings

**Figure 2:**
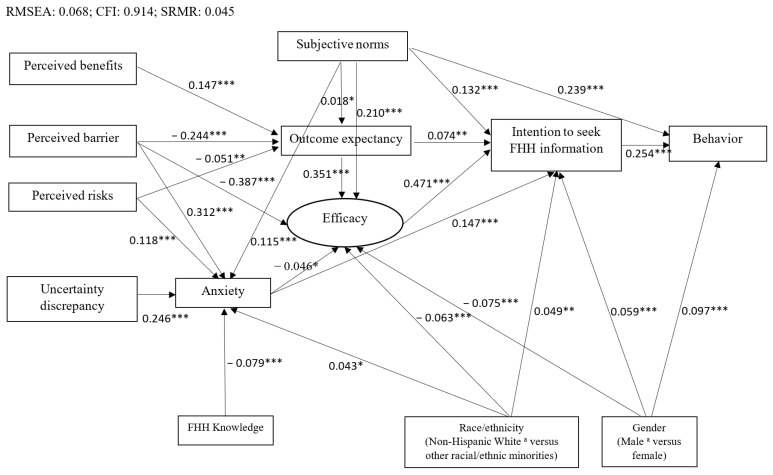
SEM model for FHH information seeking behavior among college students. *p* < 0.001 ***, *p* < 0.005 **, *p* < 0.05 *. The figure only presented the statistically significant associations (solid lines) and standardized coefficients. ^a^ Reference group. SEM: Structural equation modeling; FHH: family health history.

Figure 2 shows the final SEM findings. In particular, the SEM model fit the survey data adequately based on the model fit indices (i.e., RMSEA = 0.068; CFI = 0.914; SRMR = 0.045). Stronger intention to seek FHH information and perception of the high level of subjective norms toward FHH collection were correlated with participants’ FHH collection behavior (β = 0.254, *p* < 0.001 and β = 0.239, *p* < 0.001, respectively). Efficacy in FHH collection, anxiety associated with the uncertainty of FHH, subjective norms, and outcome expectancy of the consequences of FHH collection were significantly and positively associated with respondents’ intention to collect FHH information (β = 0.471, *p* < 0.001; β = 0.147, *p* < 0.001; β = 0.132, *p* < 0.001; and β = 0.074, *p* < 0.001, respectively). Outcome expectancy and subjective norms were positively correlated with efficacy in FHH collection (β = 0.351, *p* < 0.001 and β = 0.210, *p* < 0.001, respectively), while perceived barriers to FHH collection from family members was negatively associated with efficacy (β = −0.387, *p* < 0.001).

Perceived benefits of FHH collection and subjective norms were significantly and positively associated with outcome expectancy toward FHH collection (β = 0.147, *p* < 0.001 and β = 0.018, *p* < 0.05, respectively). However, perceived barriers to FHH collection from family members and perceived risks of developing diseases that run in a family were negatively associated with outcome expectancy (β = −0.244, *p* < 0.001 and β = −0.051, *p* < 0.005, respectively). Perceived barriers to FHH collection from family members, uncertainty discrepancy, perceived risks of getting diseases that run in families, and subjective norms were significantly and positively associated with anxiety with the uncertainty of FHH (β = 0.312, *p* < 0.001; β = 0.246, *p* < 0.001; β = 0.118, *p* < 0.001; and β = 0.115, *p* < 0.001, respectively).

### 3.4. Whether or Not Sociodemographic Characteristics and FHH Knowledge Were Correlated to FHH Collection Behavior

As shown in Figure 2, female young adults in this study were more likely to collect, and to actually collect their FHH from family members when compared to their male counterparts (β = 0.059, *p* < 0.001; β = 0.097, *p* < 0.001, respectively). Furthermore, participants who were members of racial and ethnic minority groups had a higher level of anxiety associated with lacking FHH information (β = 0.043, *p* < 0.05) and stronger intention to collect their FHH (β = 0.049, *p* < 0.005), but had lower efficacy in FHH collection (β = −0.063, *p* < 0.001) than non-Hispanic White respondents. Respondents with better FHH knowledge reported less anxiety (β = −0.079, *p* < 0.001).

## 4. Discussion

In light of the importance of FHH collection from family members among young adults, we sought to develop and test an integrated theoretical framework that could be used to examine college students’ FHH collection behavior and underlying factors using a large sample from two campuses of a public research-intensive university. Consistent with national data collected through surveying adults over the age of 18 [39] and previous research studying young adults [17], a majority of participants in our study considered collecting FHH important. However, less than half of participants (49.8%) reported actively seeking FHH information from their family members in the previous six months. Our finding is in line with previous studies. For example, a national survey of 5258 adults in the U.S. reported that only 36.9% of the respondents have actively collected their FHH [40]. Studies that were conducted to assess the use of FHH among underserved populations, such as Latinxs, African Americans, and Chinese Americans, also indicated that many of their participants had seldom collected FHH [32,41,42].

Along with a lack of FHH collection behavior from family members, we also found that our participants had low levels of FHH knowledge. In particular, only 21.8% of the respondents knew that their biological siblings are considered first-degree relatives. Moreover, about two-thirds of participants mistakenly thought that people are more similar genetically to their parents than to their brothers or sisters. This finding is aligned with a past study carried out by Rooks and Ford [43] which showed that most college students had low levels of FHH knowledge. As such, it is important to develop and implement interventions and educational programs to improve college students’ FHH knowledge and motivate them to gather FHH information from their family members.

Our SEM findings showed that young adults’ participation in FHH information collection from their family members was significantly and directly associated with their intention and likelihood to seek FHH information, and that intention to solicit FHH information was also significantly related to respondents’ efficacy in FHH communication with family members and outcome expectancy toward FHH collection. Additionally, social pressures on the participants (i.e., subjective norms) played an important role in both their FHH collection behavior and intention. Higher levels of FHH knowledge among participants was also related to lower levels of anxiety caused by FHH uncertainties. Therefore, when developing an FHH intervention and educational program, both individual factors (e.g., FHH knowledge, intention to pursue FHH information, efficacy in FHH communication, and outcome expectancy toward FHH collection) and social factors (i.e., subjective norms) should be considered. For example, a previous study has shown that family-level interventions, which take social factors into consideration, were effective in the adoption and diffusion of health behaviors [44]. As suggested, a family-based FHH intervention, which includes both young adults and other family members, may address both the individual and social factors affecting FHH collection among young adults. Given that obtaining a comprehensive and accurate FHH requires effort from all family members [44], the family unit is the ideal context for an FHH intervention.

Interestingly, our study results revealed that although racial/ethnic minority young adults exhibited both higher levels of anxiety related to unknown FHH information and more intention to gather their FHH from their family members when compared to their non-Hispanic White counterparts, they had lower efficacy in FHH collection. These findings suggested that FHH interventions and educational programs should be designed with sensitivity to the specific needs of young adult members of race/ethnicity minority groups with a goal of improving their skills, confidence and coping strategies, and reducing their anxiety levels when gathering FHH from their families. In alignment with previously published articles [19,24,32,34], female respondents in our sample were more likely to collect, and to actually collect FHH information from family members than were their male counterparts. Thus, future FHH interventions and educational programs should attempt to recruit young adult males to improve their FHH collection intention and behavior.

This study has several limitations. First, the generalizability of our findings may be limited due to the fact that participants were recruited from two campuses of one public research-intensive university. Second, this study might have a potential sample selection bias as those who responded to the survey might have had higher levels of awareness and been more interested in FHH than those who opted not to participate in our study. Third, due to the nature of cross-section surveys, we were unable to ascertain causal relationships between each construct in Figure 2. A longitudinal study is recommended in the future to examine the causal relationships. Fourth, the response rates for web-based surveys tend to be low (from 2.07% to 31.54%) in a college setting [45,46]. We employed multiple strategies to increase the response rate (e.g., providing incentives and sending three follow-up reminder emails) [47]. As expected, however, the response rate of our web-based survey was low (5.08%), but was within the range of those reported in previous studies. The possible reasons for this low response rate might be that college students did not frequently check their university email accounts, received too many emails each day, ignored messages sent from the university bulk email system, had settings on their account that sent our email directly to a spam folder, lacked interest in this study, or were busy doing their coursework and studying for their examinations [45].

Despite the above limitations, this study makes an important contribution to the limited extant research focused on understanding young adults’ FHH collection behavior and the psychological factors associated with this behavior. Consistent with the literature [17,43], our data showed that the young adults in our sample lacked FHH collection and had a deficient knowledge of FHH. Thus, it is of critical importance that FHH interventions and educational programs should be designed for and disseminated to this particular group in the future. Additionally, we created an integrated theoretical framework to examine young adults’ behavior in FHH collection, which was then tested with a large sample (over 2000 participants). The SEM findings supported the applicability of our proposed integrated theoretical framework. Psychosocial factors (i.e., intention, efficacy, outcome expectancy, subjective norms, anxiety, uncertainty discrepancy, perceived benefits, perceived barriers, perceived risks, and uncertainty discrepancy), FHH knowledge, and sociodemographic characteristics (i.e., gender, and race/ethnicity) were significantly associated with FHH collection behavior among young adults in both direct and indirect ways. Our SEM findings provide a foundation for the design and development of FHH interventions and educational programs for young adults in the future. Furthermore, it is highly recommended that these interventions and educational programs should target males, and be designed with sensitivity to the specific needs of young adult members of racial/ethnic minority and other traditionally underserved groups.

## Figures and Tables

**Table 1:** Definitions, description, examples, and data reliability and validity of the psychological constructs measured in the survey.

Constructs	Definition	Theory	# of Items	Example Question	Mean	SD	Survey Data Score Range	Theoretical Range for Each Score	Cronbach’s Alpha	Construct Validity	Interpretation
**Perceived benefits**	Perceptions of the health advantage of FHH collection	HBM	4	Knowing my FHH will help me prevent diseases/health conditions that run in my family. *[1 = Strongly disagree; 7 = Strongly agree]*	5.500	1.077	1–7	1–7	0.730	χ^2^ = 14.539, df = 1, *p* < 0.001, RMSEA = 0.076, CFI = 0.994, SRMR = 0.016	Higher score = Perceived more benefits of FHH collection
**Perceived barriers**	Beliefs concerning the actual and imagined obstacles of FHH collection from family members	HBM	13	I don’t know what questions to ask to obtain my FHH. *[1 = Strongly disagree; 7 = Strongly agree]*	2.718	1.118	1–7	1–7	0.869	χ^2^ = 847.632, df = 55, *p* < 0.001, RMSEA = 0.079, CFI = 0.935, SRMR = 0.056	Higher score = Perceived more barriers of FHH collection
**Perceived risks**	Beliefs about a likelihood of developing disease(s) that runs in family	HBM	3	How likely is it that you will get diseases that run in your family? *[1 = I definitely will not develop the diseases; 7 = I definitely will develop the diseases]*	4.304	1.155	1–7	1–7	0.753	CFA result showed a saturated model due to the three items for this construct, and all three items were significantly related to the construct (*p* < 0.001)	Higher score = Perceived more risk of developing disease(s) that runs in family
**Outcome expectancy**	The beliefs regarding the consequences of collecting FHH from family members	TMIM	3	Asking my family members about my FHH would produce ______. *[1 = A lot more negatives than positives; 7 = A lot more positives than negatives]*	5.304	1.306	1–7	1–7	0.928	CFA result showed a saturated model due to the three items for this construct, and all three items were significantly related to the construct (*p* < 0.001)	Higher score = Perceived more value on the outcomes of FHH collection
**Uncertainty discrepancy**	The gap between one’s desired and actual level of uncertainty about FHH.	TMIM	6	I know less than I would like to about my FHH. *[1 = Strongly disagree; 7 = Strongly agree]*	4.204	1.300	−2.5–6.5	−2–6.5 ^a^	0.778	χ^2^ = 16.182, df = 1, *p* < 0.001, RMSEA = 0.080, CFI = 0.995, SRMR = 0.011	Higher score = a desire for more certainty about one’s FHH
**Anxiety**	The level of anxiety associated with the uncertainty of FHH	TMIM	3	Not having as much information about my FHH as I would like makes me worried. *[1 = Strongly disagree; 7 = Strongly agree]*	3.493	1.635	1–7	1–7	0.934	CFA result showed a saturated model due to the three items for this construct, and all three items were significantly related to the construct (*p* < 0.001)	Higher score = Perceived high level of anxiety associated with uncertainty discrepancy of FHH information
**Communication efficacy ^b^**	Perceived level of skill and comfort with discussing FHH with family members	TMIM	3	I am confident that I can assess all members of my family (including those who do not live near to me) to get information of my FHH. *[1 = Strongly disagree; 7 = Strongly agree]*	4.754	1.413	1–7	1–7	0.735	CFA result showed a saturated model due to the three items for this construct, and all three items were significantly related to the construct (*p* < 0.001)	Higher score = More confidence in discussing FHH with family members
**Target efficacy ^b^**	Family members’ ability to provide an accurate FHH information.	TMIM	4	My family members would tell me everything they know about our FHH. *[1 = Strongly disagree; 7 = Strongly agree]*	5.245	1.240	1–7	1–7	0.836	χ^2^ = 31.058, df = 1, *p* < 0.001, RMSEA = 0.109, CFI = 0.993, SRMR = 0.012, and all four items were significantly related to the construct (*p* < 0.001)	Higher score = More confidence in information target’s (i.e., family members) ability to provide complete and accurate FHH information
**Coping efficacy ^b^**	Ability to cope that family members have certain FHH-related diseases	TMIM	4	Imagine that some family members became upset with you for asking them about your FHH and called you ‘nosy’. How well would you cope with this sort of reaction? *[1 = Could not cope; 7 = Could cope perfectly well]*	4.506	1.199	1–7	1–7	0.771	χ^2^ = 32.838, df = 2, *p* < 0.001, RMSEA = 0.079, CFI = 0.988, SRMR = 0.020	Higher score = More confidence in handling issues during FHH collection
**Subjective norms**	Views and influence of other people in FHH collection behavior	TPB	4	My family expects me to seek information about my FHH. *[1 = Strongly disagree; 7 = Strongly agree]*	3.555	1.542	1–7	1–7	0.904	χ^2^ = 3.832, df = 1, *p* = 0.050, RMSEA = 0.035, CFI = 1.000, SRMR = 0.004	Higher score = Perceived more social pressure from other important people regarding FHH collection
**Intention**	Likelihood of collecting FHH from family members	TPB	6	I would directly approach my family to talk about it. *[1 = Strongly disagree; 7= Strongly agree]*	4.838	1.093	1–7	1–7	0.802	χ^2^ = 3.207, df = 2, *p* = 0.201, RMSEA = 0.016, CFI = 1.000, SRMR = 0.006	Higher score = higher likelihood of collecting FHH from family members
**Behavior**	Frequency of FHH collection with family members in the past half-year	TPB	4	During the past half-year, I sought information directly about my FHH from my family members. *[1 = Never; 7 = Always]*	2.962	1.676	1–7	1–7	0.873	χ^2^ = 0.26, df = 1, *p* = 0.611, RMSEA = 0.000, CFI = 1.000, SRMR = 0.001	Higher score = higher frequent action of FHH collection from member members in the past half-year

HBM, Health Belief Model; TMIM, Theory of Motivated Information Management; TPB: Theory of Planned Behavior; χ^2^, chi-square; df, degrees of freedom; RMSEA, root mean square error of approximation; CFI, comparative fit index; SRMR, standardized root mean square residual. The internal consistency and construct validity for each construct were examined using Cronbach’s alpha and confirmatory factor analysis, respectively. Cronbach’s alpha values larger than 0.70 indicated an acceptable reliability for each construct. For construct validity, a RMSEA less than 0.08; CFI larger than 0.95; and SRMR less than 0.08 indicated an adequate model fit for each construct. ^a^ Uncertainty discrepancy was assessed by subtracting participates’ response to the question, “How much information would you like to know about your FHH?” from their answer to the question, “How much information do you know about your FHH?”, plus subtracting participants’ response to the question, “How certain do you want to be about your FHH?” from their answer to the questions, “How certain are you about your FHH?”, and plus participants’ responses to two another questions, “I know less than I would like to about my FHH.” and “I want to know more than I currently know about my FHH.” ^b^ Efficacy is a composite score of communication efficacy, target efficacy, and coping efficacy.

**Table 2:** FHH knowledge among college students in our sample.

Conceptual Knowledge Items	Correct (%)
FHH tells you which diseases you will certainly develop. (False)	70.6%
If you have a FHH of a disease, you are more likely to get the disease yourself. (True)	84.6%
It is important to know how old your relatives were when they were diagnosed with cancer. (True)	77.2%
You can only inherit breast cancer from your mother’s side of the family. (False)	66.9%
People are genetically more similar to their parents than to their brothers or sisters. (False)	25.9%
In terms of FHH, my biological brothers and sisters are considered my second-degree relatives. (False)	21.8%
Averagely	57.8%

FHH: family health history. Note: We adopted the knowledge items from three studies [24,25,32].

## Data Availability

As a data sharing strategy was not included in the original application for institutional review board review, study data are not publicly available.
